# Cumulative live-birth, perinatal and obstetric outcomes for POSEIDON groups after IVF/ICSI cycles: a single-center retrospective study

**DOI:** 10.1038/s41598-020-68896-1

**Published:** 2020-07-16

**Authors:** Raed K. Abdullah, Nenghui Liu, Yuhao Zhao, Yang Shuang, Zhang Shen, Hong Zeng, Jielei Wu

**Affiliations:** Reproductive Medical Center, Xiangya Hospital, Central South University, 87 Xiangya Road, Changsha City, 410008 Hunan Province People’s Republic of China

**Keywords:** Health occupations, Medical research

## Abstract

Recently, perinatal outcomes and cumulative live birth rate (CLBR) have widely been utilized to assess the fertility outcomes and safety of assisted reproductive technology (ART), but more robust research is needed to address the success rates of live-healthy births resulting from this procedure, particularly for patients with low prognosis. This study aims to assess and comparative perinatal outcomes and CLBR per cycle of in vitro fertilization/Intracytoplasmic sperm injection (IVF/ICSI) between four groups of low prognosis characterized by POSEIDON criteria. A retrospective assessment was done among infertile women with a low prognosis undergoing IVF/ICSI at a reproductive center in China. Data were collected between January 2011 and December 2015 with a follow-up of at least two years, and censoring was defined by three-cycle completion, discontinuation, or having a live birth. Participants were grouped into 4 groups according to the POSEIDON classification (POSEIDON1, POSEIDON2, POSEIDON3, and POSEIDON4). The main outcomes were perinatal and obstetric outcomes with CLBR per cycle after IVF/ICSI procedure. And IVF/ICSI-technique outcomes as a secondary outcome. Statistical analyses were performed by SPSS, and a *p* value of < 0.05 was considered significant. A total of 461 eligible participants underwent a total of 825 IVF/ICSI cycles. POSEIDON1 had the best perinatal outcomes in terms of live births (≥ 28w) (54.8%). POSEIDON4 had a higher risk for perinatal and obstetric complications with abortion rate (9.8%); LBW (11.7%), PTD (23.5%), PROM (11.7%), and gestational diabetes (17.6%). POSEIDON2 had a high rate for malpresentation (14.2%), and cesarean delivery(57.2%), while POSEIDON3 was much associated with the occurrences of placenta previa (9.3%) compared to other groups (*p* value = 0.001). After adjusting odds ratio by age and BMI, POSEIDON4 had the least odds for biochemical pregnancy (*p* value = 0.019); and the least odds for clinical pregnancy (*p* value = 0.001) of the four groups. CLBR per cycle was better in POSEIDON1 and increased with an increasing number of cycles in all groups during the three cycles. Conservative CLBR after three complete cycles were 77.27%, 42.52%, 51.4% and 22.34%, while optimistic CLBR were 79.01%, 51.19%, 58.59% and 34.46% in POSEIDON1 to POSEIDON4, respectively. Younger women with low prognosis and normal ovarian reserve have a higher probability for live births and better perinatal outcomes compared with older women with poor or normal ovarian reserve. Besides, young women with low prognosis, despite ovarian reserve status, can increase their probability of conception and get relatively higher CLBR by undergoing multiple cycles of IVF/ICSI. Age is therefore considered as a critical parameter in predicting the perinatal outcome and CLBR.

## Introduction

Infertility accounts for the majority of gynecological conditions worldwide, and its treatment has an overwhelmingly monetary and time concern^1,2^. These concerns resonate with couples’ dire need for a child^3^. Although not always successful, IVF is known to have revolutionized in infertility burden. In recent decades, the rate of conceiving after the IVF technique could reach up to 60% among young couples^4^.

In a typical IVF cycle, the number of oocytes aspirated constitutes a vital step which also defines ovarian response and greatly determines fertility outcome^5^. Initially defined by retrieval of 4–9 oocytes after conventional stimulation of ovary, poor ovarian response’s (POR)^6,7^ definition evolved with time to not only considering oocyte retrieval factor but also advanced maternal age (i.e. > 40) and poor ovarian reserve and history of POR. The latter form a basis for BOLOGNA criteria^8^. An estimate of 9–24% of women undergoing IVF are poor ovarian responders^9^.

Even though antral follicle count (AFC) and anti-Müllerian hormone (AMH) are currently regarded to be good predictors of ovarian reserve, the rate of age-related blastocyst/embryo aneuploidy plays a vital role in the management of low prognostic women^10^. Following reported setbacks in BOLOGNA criteria, POSEIDON (Patient-Oriented Strategies Encompassing Individualized Oocyte Number) criteria were further developed to refine the definition of low prognosis, depending on age with AMH^11^ and AFC, and offering clinicians clinical guidance to conducting ART^12–14^.

The success of an IVF cycle strongly depends on ovarian response to gonadotrophin stimulation, amid other factors^15^. Not only does suboptimal ovarian response associated with poor success rate after IVF cycles, but it also is associated with a higher risk of obstetric and perinatal complications in resulting pregnancies^16^ as compared to other modalities such as intrauterine insemination^17^. Moreover, the procedure comes at a high price and costs time^17^. Despite the aforementioned setbacks, that nearly make IVF a decision of exclusion, infertile couples opting for IVF would need to weigh the setbacks against anticipated outcomes and success rates for live birth. This urges deep exploration and robust researches in the subject matter. It follows that, since POSEIDON criteria were introduced within the last decade, several publications have opted to assess different fertility outcomes among the four groups of low prognosis defined by the criteria^18,19^. With the majority assessing the cumulative probability of pregnancies^20,21^, a few assessed cumulative probabilities of pregnancy. However, to our knowledge, the long term perinatal and obstetric outcome is still unexplored among poor ovarian responders stratified by the criteria. Our study, therefore, aimed to investigate the perinatal and obstetric outcomes with CLBR per cycle after IVF/ICSI procedure between women with low prognosis characterized by POSEIDON criteria as a main outcomes. And IVF/ICSI-technique outcomes as a secondary outcome.

## Results

**Figure 1:**
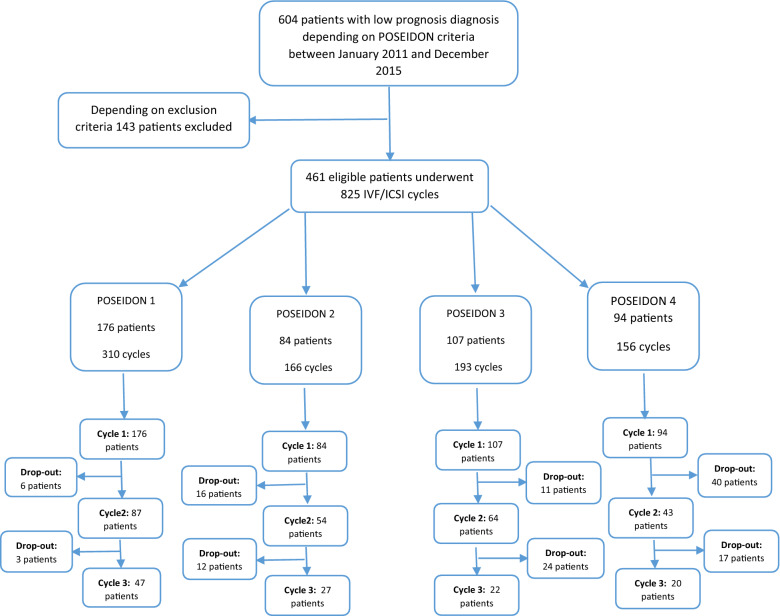
IVF/ICSI patients’ flow diagram (drop-out: patients who abstain from scheduled follow-up visits at our center during the period of our study).

Medical records of 604 patients with low prognosis were reviewed from January 2011 to December 2015 at Xiangya Hospital. A total of 461 women underwent one, two, or three cycles (all were 825 cycles) of IVF/ICSI were eligible for inclusion in the study. A total of 176, 84, 107, and 94 were classified as POSEIDON 1, 2, 3, and 4 respectively. These patients had undergone a total of 310, 166, 193, and 156 IVF/ICSI cycles, respectively, Fig. 1.

The obstetrical history and demographic characteristics in Table 1 show that in POSEIDON1 and POSEIDON3 more than two-thirds of the participants had normal BMI (< 25 kg/m^2^) and no participant was recorded to be obese, the results reached statistical significance (*p* value = 0.00). Less than a third of participants in POSEIDON1 reported irregular menses with the majority reporting regular (*p* value = 0.00). POSEIDON4 and POSEIDON2 had the highest mean of previous abortion and ectopic pregnancy of all four groups, however, this result did not reach statistical significance (*p* value = 0.492). Regarding the number of previous spontaneous abortions, all four groups had more or less similar results (*p* value = 0.158). POSEIDON2 and POSEIDON4 had higher mean infertility durations and the difference reached statistical significance (*p* value = 0.00). It should be mentioned that these features may be concerning the characteristics of the POSEIDON groups. Regarding causes of infertility, the most were either female (higher in POSEIDON1, 81.8%) or both female and male (higher in POSEIDON2, 45.2%), (*p* value = 0.00), but few numbers were recorded as male cause alone or rather unexplained. None of the women in this study were reported to smoke or drink. Also, no one had previous ovariectomy or previous chemotherapy.

**Table 1:** Obstetrical history with demographic data in the first IVF/ICSI cycle.

		POSEIDON 1 (N = 176)	POSEIDON 2 (N = 84)	POSEIDON 3 (N = 107)	POSEIDON 4 (N = 94)	*p* value
Age (years)^a^		28 (26–31)	38 (37–38)	29 (28–30)	37 (36–38.75)	**0.00**
BMI N (%)^b^	Normal (< 25)	157 (89.2%)	64 (76.2%)	89(83.2%)	64 (68%)	**0.00**
		*0.1370*	*0.5490*	*0.8505*	***0.000***	
	Overweight (25–30)	19 (10.8%)	20 (23.8.8%)	18 (16.8%)	30 (32%)	
		*0.1370*	*0.5490*	0.8505	***0.000***	
	Obese (> 30)	0 (0%)	0 (0%)	0 (0%)	0 (0%)	
Menstrual cycle in general N (%)^b^	Regular	160 (91%)	58 (69%)	88 (82.2%)	74(78.7%)	**0.00**
		***0.00308***	*0.00637*	*0.98587*		
	Irregular	16 (9%)	26 (31%)	19 (17.8%)	20 (21.3%)	
		***0.00308***	*0.00637*	*0.98587*		
No. previous pregnancy N (%)^b^	Primiparous	108 (61.3%)	24 (28.6%)	58 (54.2%)	39(41.5%)	**0.00**
	Multiparous	68 (38.4%)	60 (71.4%)	49 (45.8%)	55(58.5%)	
Previous abortions^a^		1 (0–1)	2 (1–3)	1 (0–2)	2 (1–3)	0.492
Previous spontaneous abortion^a^		0 (0–0)	0 (0–0)	0 (0–1)	0 (0–0)	0.158
Previous ectopic pregnancy^a^		0 (0–0)	0 (0–0)	0 (0–0)	0 (0–0)	0.734
Infertility type N (%)^b^	Primary	56 (31.8%)	29 (34.5%)	36 (33.6%)	40(42.5%)	0.259
	Secondary	120 (68.2%)	55 (65.5%)	71 (66.4%)	54(57.5%)	
Infertility duration (years)^a^		3 (2–5)	7 (6–9)	3 (2–5)	8 (6–10)	**0.00**
Infertility cause N (%)^b^	Female	144 (81.8%)	41(48.8%)	77 (71.9%)	51(54.2%)	**0.00**
		*0.06714*	***0.00063***	*0.00548*	*0.03929*	
	Male	8 (4.5%)	5 (5.9%)	2 (1.85%)	6 (6.3%)	
	Male and female	22 (12.5%)	38 (45.2%)	27 (25.2%)	35(37.2%)	
		*0.0336*	***0.0001***	*0.0096*	*0.0929*	
	Unexplained	2 (1.1%)	0 (0)	1 (0.9%)	2 (2.1%)	

^a^Median test, data presented as median (interquartile range); ^b^Pearson’s Chi-square. Data are mean ± standard deviation or % unless otherwise specified; Italics are post-hoc *p* values after Bonferroni adjustment and values < 0.00625 were statistically significant; bolded are statistically significant *p* values.

In Table 2 we compare IVF/ICSI cycle parameters among the four groups. POSEIDON1 had the highest recorded of AFC, AMH (*p* value = 0.00). Values basal FSH levels and basal LH levels did not reach statistical significance among the four groups (*p* value = 0.297 and 0.349 respectively). Furthermore, basal E2 was the highest recorded in POSEIDON1 followed by POSEIDON3, with POSEIDON2 ranking the least, and the difference reached statistical significance (*p* value = 0.002), but mean basal progesterone levels did not reach statistical significance (*p* value = 0.316). POSEIDON1 showed the highest levels of LH, E2, and progesterone on trigger day (*p* value = 0.04 , 0.016, and 0.00 respectively), while POSEIDON4 ranked the least. Furthermore, no significant differences were found in the total days and total dose of using all medicaments between the 4 groups. The follicles ≥ 14 mm on trigger day was better in POSEIDON1 and POSEIDON3, reaching statistical significance (*p* value = 0.018). There was no statistically significant difference between frozen and fresh embryo transfers among the four groups. None of the patients recorded the ovarian hyperstimulation syndrome (OHSS) diagnosis.

**Table 2:** Comparison IVF/ICSI-technique parameters between four groups throughout the IVF/ICSI course.

	POSEIDON1 (N = 310 cycles) (Mean ± SD)	POSEIDON 2 (N = 166 cycles) (Mean ± SD)	POSEIDON 3 (N = 193 cycles) (Mean ± SD)	POSEIDON4 (N = 156 cycles) (Mean ± SD)	*p* value
AFC (antral follicle count)^a^	9 (8–9)	8 (7–9)	1.2 (1–2)	1.8 (1–2)	**0.00**
AMH (ng/ml)^a^	5.7 (4.3–7.13)	3.12 (2.22–3.505)	1.2 (0.5–1.93)	1.1 (0.22–1.31)	**0.00**
Basal FSH (mIU/mL)^a^	6.34 (5.61–8.36)	8.6 (6.8–9.76)	7.19 (6.39–9.36)	8.04 (6.53–9.70)	0.297
Basal LH (mIU/mL)^a^	3.7 (2.7–4.65)	4.25 (3.2–5.65)	3.94 (2.97–5.06)	4.17 (3.09–5.74)	0.349
Basal Estradiol (E2) (ng/mL)^a^	38.45 (33.28–44.95)	33.80 (30.80–45.60)	38.39 (33.54–48.75)	30.46 (28.02–33.73)	**0.002**
Basal Progesterone (ng/mL)^a^	0.77 (0.53–0.85)	0.74 (0.56–0.82)	0.77 (0.51–0.87)	0.74 (0.50–0.83)	0.316
LH on trigger day (mIU/mL)^a^	0.77 (0.62–0.88)	0.71 (0.45–1.05)	0.64 (0.45–0.96)	0.66 (0.53–0.97))	**0.004**
Estradiol on trigger day (E2) (ng/mL)^a^	2,526 (2061.50–2,713.50)	2,256 (1,478.00–2,782.50)	2,366 (1,456.50–3,698.00)	2,205 (2,116.25–2,611.75)	**0.016**
Progesterone on trigger day(P) (ng/mL)^a^	1.76 ± 4.10	0.87 ± 1.06	1.66 ± 3.27	0.98 ± 1.09	**0.00**
rFSH starting dose (IU)^b^	224.01 ± 55.34	227.01 ± 56.98	215.51 ± 65.60	214.22 ± 67.97	0.438
rFSH total dose (IU)^b^	2098.84 ± 844.56	2,201.60 ± 903.87	2067.053 ± 1,084.89	2,113.24 ± 1,043.70	0.564
HMG duration (days)^b^	5.13 ± 2.92	5.72 ± 2.62	5.20 ± 2.76	5.45 ± 2.94	0.502
HMG dose (IU)^a^	75 (75–75)	75 (75–75)	75 (75–75)	75 (75–75)	–
HMG total dose (IU)^a^	75 (15–525)	225 (187–675)	225 (187.50–675.00)	187 (45–525)	0.433
GnRH agonist start dose (mg)^a^	0.1 (0.1–0.5)	Constant Value	0.1 (0.1–0.5)	0.1 (0.1–0.5)	0.392
GnRH agonist duration (days)^a^	7 (6.00–12.00)	Constant value	6.5 (6.00–12.00)	7.0 (5.00–12.00)	0.654
GnRH agonist total dose (mg)^a^	0.6 (0.6–0.6)	Constant value	0.6 (0.6–0.6)	0.6 (0.5–0.6)	0.161
GnRH antagonist start dose (mg)^c^	Constant value	Constant value	Constant value	Constant value	1.00
GnRH antagonist total dose (mg)^a^	Constant value	Constant value	Constant value	Constant value	0.154
GnRH antagonist duration (days)^a^	Constant value	Constant value	Constant value	Constant value	0.449
GH duration (days)^a^	6.00 (6.00–6.00)	Constant value	6.00 (6.00–6.00)	6.00 (6.00–6.00)	0.112
CC total dose (mg)^a^	25 (5–45)	35 (25–45)	40 (20.50–45.00)	25 (5.00–45.00)	0.685
CC duration (days)^a^	8 (5.00–8.00)	6.5 (5.00–9.00)	8.00 (5.75–9.00)	7.50 (5.00–8.00)	0.894
Duration of stimulation (days)^a^	8 (8–10)	8 (8–10)	9 (8–10)	8 (8–10)	0.727
Number of follicles ≥ 14 mm on trigger day^a^	6 (4–5)	2 (3–5)	4 (3–5)	2 (3–5)	**0.018**

^a^Median test, data presented as median (interquartile range); ^b^one-way ANOVA; data are mean ± standard deviation or % unless otherwise specified; bolded are statistically significant *p* values.

Table 3 shows the vital outcome parameters along with IVF/ICSI procedure. POSEIDON1 and 3 displayed the better mean numbers of oocytes retrieved, number metaphase II Oocytes, usable embryos, and high-quality embryos obtained on day 3 of the four groups (*p* value = 0.00). Regarding pregnancy outcomes following IVF/ICSI, a total of 427 (51.7%) and 301(36.5%) biochemical and clinical pregnancies were recorded, respectively, among all the four groups. Nearly half of all biochemical and clinical pregnancies were recorded in POSEIDON1 (44% and 51.5% respectively) while POSEIDON4 recorded the least (12.6% and 10.30% respectively). Regarding delivery outcomes, of all 248 (30%) (227 singleton and 21 twins) deliveries recorded among four groups, POSEIDON1 constituted the majority, POSEIDON3 ranked second, POSEIDON2 ranked third, and POSEIDON4 was the least for singleton and twins pregnancy.

**Table 3:** Comparison of IVF/ICSI-technique outcomes.

		POSEIDON1 (N = 310 cycles)	POSEIDON2 (N = 166 cycles)	POSEIDON3 (N = 193 cycles)	POSEIDON4 (N = 156 cycles)	Total (%)	*p* value
IVF/ICSI Technique’s outcome Mean ± SD	No. Oocytes retrieved	5.82 ± 1.48	3.26 ± 1.86	4.32 ± 1.20	3.51 ± 2.54	**Na**	**0.00**
	No. metaphase II Oocytes	2.31 ± 1.34	1.86 ± 1.43	1.87 ± 2.20	1.44 ± 1.40	**Na**	**0.00**
	No. Embryos Obtained (usable embryo)	2.22 ± 0.83	1.85 ± 0.61	2.05 ± 1.29	2.09 ± 1.48	**Na**	**0.00**
	No. high quality Embryos Obtained in day 3	1.37 ± 1.28	1.06 ± 0.78	1.22 ± 0.90	0.93 ± 0.73	**Na**	**0.00**
	No. Embryo transferred (Fresh)	0.89 ± 0.57	0.83 ± 0.57	0.82 ± 0.65	0.86 ± 0.68	**Na**	0.572
	No. Embryo transferred (Frozen)	0.27 ± 0.56	0.29 ± 0.52	0.40 ± 0.67	0.24 ± 0.57	**Na**	0.069
Pregnancy’s outcome N (%)	Biochemical pregnancy	188(44%)	85(19.9%)	100 (23.4%)	54 (12.6%)	427 (100%)	**0.00**
		***0.03404***	*0.81108*	*0.99937*	*0.10612*		
	Clinical Pregnancies	155(51.5%)	51(16.9%)	64 (21.3%)	31 (10.3%)	301 (100%)	**0.00**
		***0.01339***	*0.97251*	*0.07780*	*0.29178*		
Delivery (live births per cycle) N (%)	Singleton	129 (56.8%)	32 (14.1%)	49 (21.6%)	17 (7.5%)	227 (100%)	**0.00**
	Twins	7 (33.3%)	4 (19.1%)	6 (28.6%)	4 (19%)	21 (100%)	0.021

*NA* not applicable, statistical test: Kruskal–Wallis Chi-square; italics are post-hoc *p* values after Bonferroni adjustment and values < 0.00625 were statistically significant; bolded are statistically significant *p* values.

Table 4 illustrates the comparison of the perinatal and obstetric outcomes in a single pregnancy among the four POSEIDON groups. Of all 227 singleton live births recorded at ≥ 28 weeks, POSEIDON1constituted the majority (29 live birth, 42.6%), and the finding reached statistical significance (*p* value = 0.00). Moreover, all four groups showed fairly similar recorded mean birthweights and gestational ages (*p* value = 0.983 and 0.228 respectively). Although none of all live births were recorded to have malformations. POSEIDON4 had the highest probability (5.8%) of all 9 live-births who were reported to be SGA, (*p* value = 0.912). Besides, POSEIDON4 recorded the highest probability for LBW (11.7%) across the four groups and was also statistically significant (*p* value = 0.00). Interestingly, none of the four groups recorded LGA. In Table 4 also we illustrate the obstetric outcomes and found that of all 59 histories of abortion recorded across the groups, POSEIDON4 revealed followed by POSEIDON2 the highest probability for abortion (9.8% and 9.2% respectively), and placenta previa (9.3% and 6.1% respectively) as compared to other groups, the differences reached statistical significance (*p* value = 0.001). POSEIDON3 showed the highest probability for early PTD (< 32 weeks) (6.2%), pre-eclampsia was higher in POSEIDON4 (5.8%), however, none of these findings reached statistical significance. Moreover, a total of 117 and 110 normal vaginal delivery and cesarean section deliveries were recorded, respectively, POSEIDON1 had the highest probabilities for having normal vaginal delivery (55.8%) (*p* value = 0.001). On the other side, POSEIDON2 recorded the highest probabilities for cesarean section and malpresentation rate (14.2% and 57.2% respectively) (*p* value = 0.001) as compared to other groups. POSEIDON4 showed the highest probability for PTD (23.5%), PROM (11.7%), gestational diabetes (17.6%) and ectopic pregnancies (2.6%), and all except ectopic pregnancy reached statistical significance (*p* value = 0.001). Placenta abruption was noticed in POSEIDON2 (2%) and POSEIDON1 (0.7%) without statistical significance. Only one stillbirth was recorded in POSEIDON2 (2%).

**Table 4:** Perinatal and obstetric outcomes per singleton pregnancy comparison.

	POSEIDON 1 (N = 303 cycles)	POSEIDON 2 (N = 162 cycles)	POSEIDON 3 (N = 187cycles)	POSEIDON 4 (N = 152 cycles)	*p* value
Singleton live birth (≥ 28w) N (%)^b^ Number N (%)	129 (42.6%)	32 (19.7%)	49 (26.2%)	17 (11.1%)	**0.00**
Birthweight (g) ^a^Mean ± SD	3,051.84 ± 355.31	3,058.16 ± 317.44	3,090.63 ± 276.63	3,091.18 ± 322.70	0.983
Gestational age (weeks)^a^ Mean ± SD	37.25 ± 1.32	37.14 ± 1.71	36.88 ± 1.52	36.94 ± 1.56	0.228
SGA N (%)^b^	5 (3.8%)	2 (4%)	1 (3.1%)	1 (5.8%)	0.912
LGA N (%)^b^	0 (0)	0 (0)	0 (0)	0 (0)	N/A
LBW N (%)^b^	7 (5.4%)	3 (6.1%)	2 (6.2%)	2 (11.7%)	**0.00**
Malformations N (%)^b^	0 (0)	0 (0)	0 (0)	0 (0)	N/A
Mode of delivery N (%)^b^					
Normal vaginal delivery	72 (55.8%)	21 (42.8%)	17 (53.1%)	7 (41.2%)	**0.001**
	***0.0000***	*0.58015*	*0.72466*	*0.00023*	
Cesarean	57 (44.2%)	28 (57.2%)	15 (46.9%)	10 (58.8%)	
	***0.0000***	*0.58015*	*0.72466*	*0.00023*	
PTD (< 37 weeks) N (%)^b^	24 (18.6%)	10 (20.4%)	6 (18.7%)	4 (23.5%)	**0.001**
Early PTD (< 32 weeks) N (%)^b^	4 (3.1%)	2 (4%)	2 (6.2%)	1 (5.9%)	0.933
PROM N (%)^b^	8 (6.2%)	4 (8.1%)	2 (6.2%)	2 (11.7%)	**0.001**
Pre-eclampsia N (%)^b^	3 (2.3%)	2 (4%)	1 (3.1%)	1 (5.8%)	0.952
	1 (0.7%)	1 (2%)	0 (0.0%)	0 (0.0%)	0.782
	3 (2.3%)	3 (6.1%)	3 (9.3%)	1 (5.8%)	**0.001**
Gestational diabetes N (%)^b^	6 (4.6%)	4 (8.1%)	3 (9.3%)	3 (17.6%)	**0.001**
Malpresentation N (%)^b^	16 (12.4%)	7 (14.2%)	1 (3.1%)	1 (5.8%)	**0.001**
Stillbirth/all cycles N (%)^b^	0 (0.0%)	1 (0.6%)	0 (0.0%)	0 (0.0%)	0.345
Abortion/all cycles N (%)^b^	15 (4.9%)	15 (9.2%)	14 (8.6%)	15 (9.8%)	**0.001**
Ectopic pregnancy/all cycles N (%)^b^	5 (1.7%)	4 (2.1%)	3 (1.6%)	4 (2.6%)	0.071

^a^Kruskal–Wallis Chi-square; ^b^Pearson’s Chi-square, *SGA* small for gestational age, *LBW* low birth weight, *LGA* large for gestational age (> 90%) (≥ 4,500 g), *PTD* preterm delivery, *PROM* premature rupture of membrane; bolded are statistically significant *p* values; italics are post-hoc *p* values after Bonferroni adjustment and values < 0.00625 were statistically significant; bolded are statistically significant *p* values.

Regression analysis was done utilizing obstetric and perinatal outcomes identified in Table 4. Parameters were adjusted to participant's age and BMI and results are shown in Table S1 (Supplementary file), (only parameters with statistical significance shown). POSEIDON4 was used as a reference group. POSEIDON4 were more likely to have PTD (< 37 weeks) as compared to POSEIDON1, however, there was no statistical significance after adjusting the finding to age and BMI, AOR of 4.157 (CI 0.892–19.374). There were no statistically significant differences between POSEIDON2 or 3 versus POSEIDON4 in terms of PTD (< 37 weeks). POSEIDON2 participants were more likely to have fetal malpresentationas compared to POSEIDON4 with COR and AOR of 9.274 (CI 1.210–71.088) and 12.427 (1.062–145.470), respectively. There was no statistically significant difference between POSEIDON2 and 3 versus POSEIDON 4 in terms of fetal malpresentation. POSEIDON1 were more likely to have biochemical pregnancy as compared to POSEIDON4 with COR and AOR of 2.302 (CI 1.368–3.872) and 3.540 (CI 1.421–8.815), respectively. POSEIDON2 participants were more likely to have biochemical pregnancy as compared to POSEIDON4 with COR and AOR of 1.542 (CI 0.875–2.716) and 1.520 (CI 0.855–2.700), respectively. POSEIDON3 participants were more likely to have biochemical pregnancy as compared to POSEIDON4 with COR and AOR of 1.298 (CI 0.716–2.355) and 1.938 (CI 0.777–4.839), respectively.

Moreover, POSEIDON1 were more likely to have clinical pregnancy as compared to POSEIDON4 with COR and AOR of 2.154 (CI 1.274–3.641) and 4.049 (CI 1.639–10.000), respectively. POSEIDON2 participants were more likely to have clinical pregnancy as compared to POSEIDON4 with COR and AOR of 1.588 (CI 0.883–2.856) and 1.633 (CI 0.900–0.963), respectively. POSEIDON3 participants were more likely to have clinical pregnancy as compared to POSEIDON4 with COR and AOR of 0.853 (CI 0.458–1.590) and 1.538 (CI 0.612–3.866), respectively.

**Figure 2:**
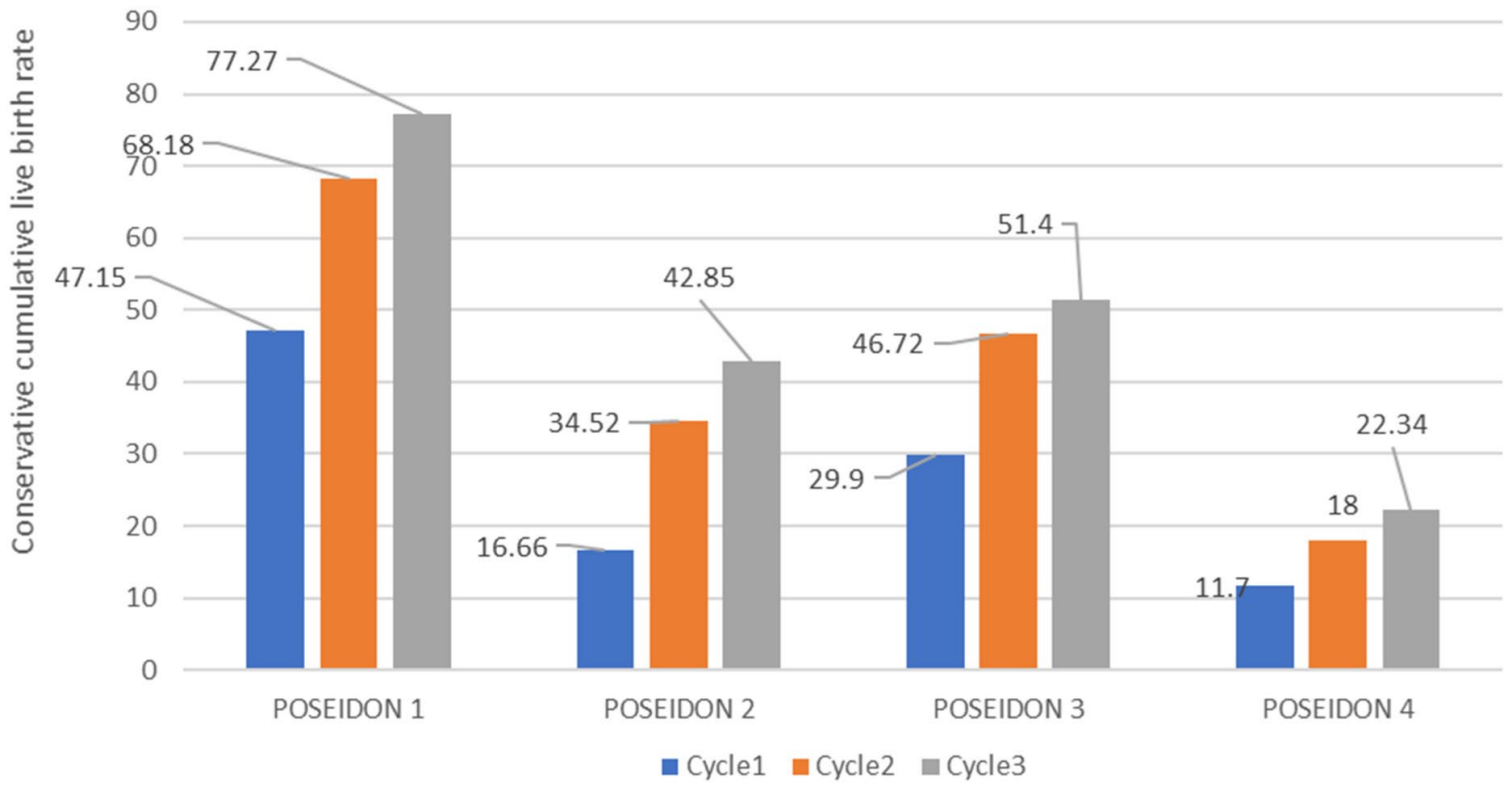
The conservative CLBR for low prognosis.

**Figure 3:**
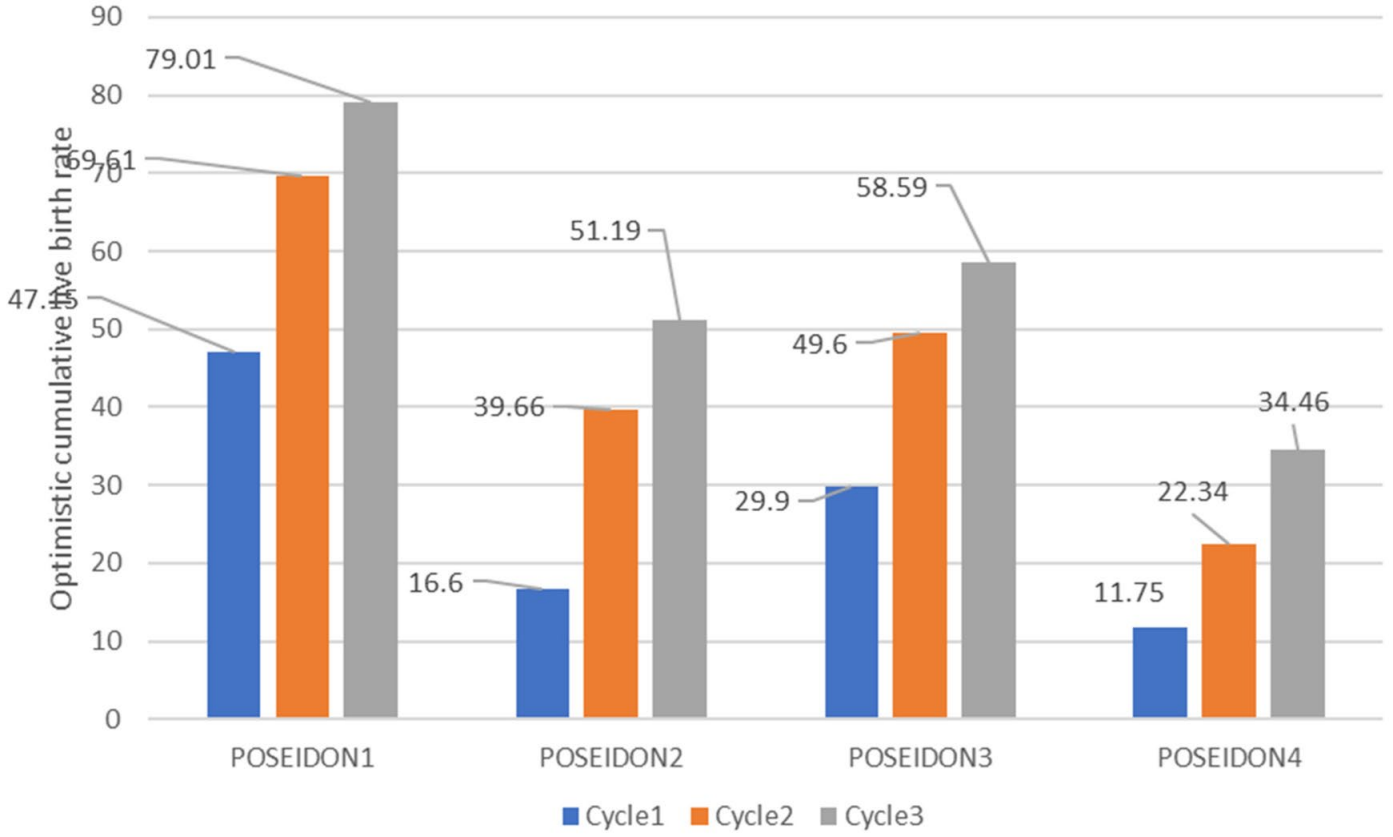
The optimistic CLBR for low prognosis.

**Figure 4:**
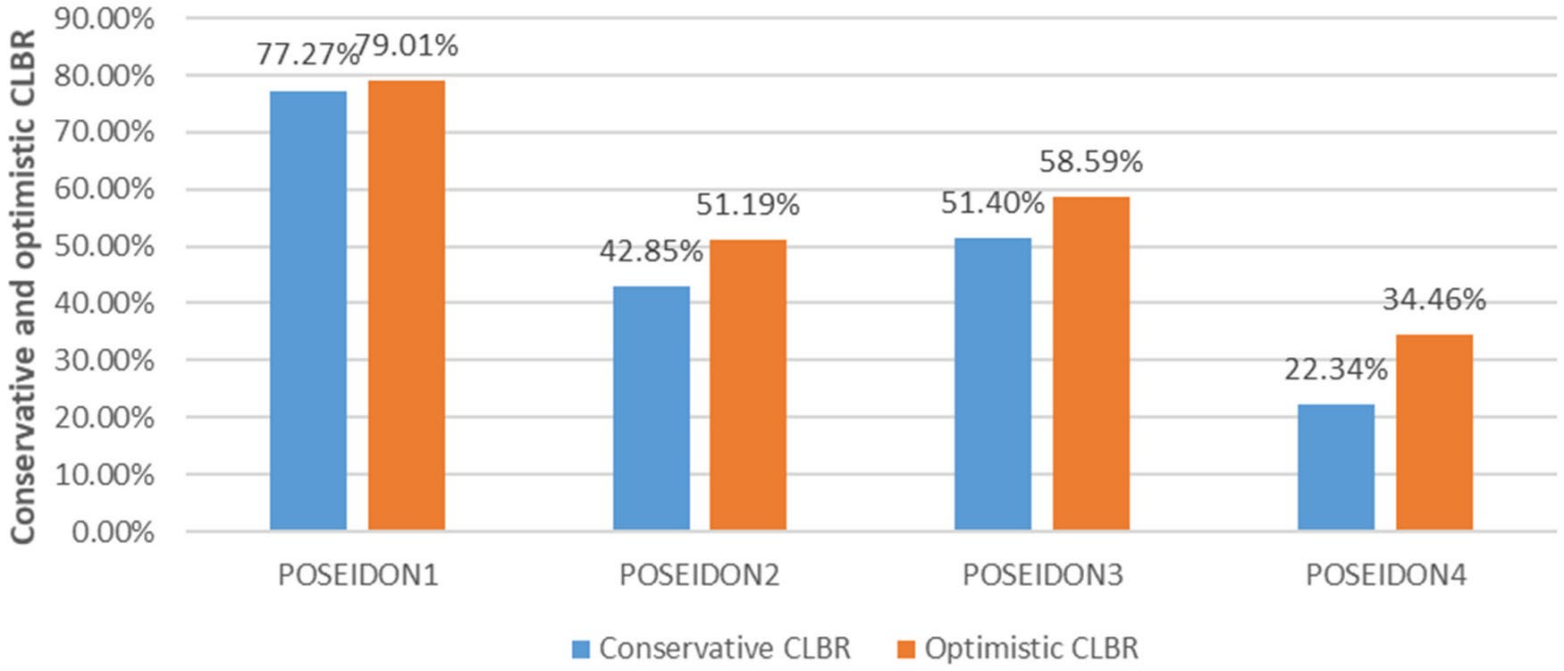
The optimistic and conservative CLBR for low prognosis after three cycles.

Table 5 illustrates the optimistic and conservative CLBR among four POSEIDON groups in each of the three cycles. It can be noticed that of all CLBR recorded in POSEIDON1, 2, 3, and 4, the majority were recorded in POSEIDON1 as compared to the other groups and cycles for conservative and optimistic CLBR as illustrated in Figs. 2 and 3 respectively. Figure 4 shows conservative CLBR per cycle after three complete cycles (77.27%, 42.52%, 51.4% and 22.34%) and optimistic CLBR (79.01%, 51.19%, 58.59% and 34.46%) in POSEIDON1 to POSEIDON4 respectively.

**Table 5:** Optimistic and conservative CLBR per cycle.

Cycle	Number of patients (N)	Live birth, N (%)	Did not return for subsequent Cycle (N)	CLBR across all cycles (%)	
				Conservative	Optimistic
POSEIDON 1					
Cycle1	176	83 (47.2%)	–	47.15	47.15
Cycle2	87	37 (42.5%)	6	68.18	69.61
Cycle3	47	16 (34%)	3	77.27	79.01
POSEIDON 2					
Cycle1	84	14 (16.6%)	–	16.66	16.6
Cycle2	54	15 (27.7%)	16	34.52	39.66
Cycle3	27	7 (25.9%)	12	42.85	51.19
POSEIDON 3					
Cycle1	107	32 (39%)	–	29.9	29.9
Cycle2	64	18 (28.1%)	11	46.72	49.60
Cycle3	22	5 (22.7%)	24	51.4	58.59
POSEIDON 4					
Cycle1	94	11 (11.7%)	–	11.7	11.75
Cycle2	43	6 (13.9%)	40	18	22.34
Cycle3	20	4 (20%)	17	22.34	34.46

## Discussion

Women with low prognosis after ART treatment can be stratified by using the POSEIDON criteria. Despite many studies have reported the CLBR among POSEIDON groups^19,22,23^, to our knowledge, the long term perinatal and obstetric outcome is still unexplored among women with low prognosis stratified by the POSEIDON criteria. In this study, a total of 461 patients with low prognosis underwent 825 complete IVF/ICSI cycles, of which 176 (310 cycles), 84 (166 cycles), 107 (193 cycles), and 94 (156 cycles) were in POSEIDON 1, 2, 3, and 4 respectively.

From the demographic characteristics and obstetric history, the majority of participants were found to have regular menses with highest mean for POSEIDON1, with normal BMI or overweight. Knowing that obesity affecs the IVF outcomes^24^. Out of the four groups, POSEIDON4 and POSEIDON2 had the highest mean of previous abortion but didn’t obtain statistical significance. This could be explained by three decades, but recently abolished, one-child policy in China^25,26^.

Regarding the hormonal tests, POSEIDON1 was observed to have the highest median levels of AMH and AFC. In contrast, Shi et al.^22^ reported a higher AFC in POSEIDON2. The conflicting findings were attributed to the different methodological approaches in stratification by POSEIDON criteria. Unlike our study, Shi et al. grouped participants into five strata adding the history of previous stimulation and oocytes retrieved into the equation. In this study, we noticed that basal E2 was highly recorded in POSEIDON1 followed by POSEIDON3, with POSEIDON2 ranking the least, and the difference reached statistical significance. This result can illustrate because the estrogen levels rise, during the follicular phase, in parallel to the growth and number of a dominant follicle^27^. This coincides that the number of follicles (≥ 14 mm) on trigger day was better in POSEIDON1 followed by POSEIDON3. In addition, POSEIDON1 followed by POSEIDON3 displayed the highest mean numbers of the vital outcome parameters of the IVF/ICSI procedure and biochemical pregnancies as compared to other groups.

Regarding perinatal and obstetric outcomes our study utilized the total number of live births of singleton pregnancy as a denominator, as previously suggested^28^. We found that the pregnancy outcomes in POSEIDON1 constituted the majority of all clinical pregnancies in the entire 227 singleton deliveries recorded, followed by POSEIDON3, and POSEIDON2, while POSEIDON4 contained the least. This result could be explained by the fact that POSEIDON1 and 3 had the best of IVF/ICSI technique’s outcomes, and the number of these measures was higher in POSEIDON1and 3 than POSEIDON2 and 4. These results harmonize with the previous study^22^. The findings were also statistically significant after adjusting for age and BMI. It is an interest that none of the four groups attained statistical significant difference in terms of recorded mean birthweights and gestational ages. As there was no statistically significant difference between frozen and fresh embryo transfers among the four groups, our mean birthweights results contradicted with those of Sazonova et al.^28^ who reported higher rates of large for gestation age in fresh embryo transfer. For SGA and LBW, POSEIDON4 had the highest probability across the four groups, in LBW reached statistical significance. Regarding low birth weight, our findings coincided with those of Wennerholm et al*.*^29^. POSEIDON4 followed by POSEIDON2 revealed the highest probability for abortion. In general, the previous study reported an increase in the rate of miscarriage after IVF, from 43.1% in a younger age to 65.2% in older age^30^. In POSEIDON1 the patients had the highest probabilities for having a normal vaginal delivery, while in POSEIDON2 most patients had cesarean section and were accompanied with a high rate of malpresentation in this group. It can, however, be noticed, in all groups that caesarian delivery was higher than vaginal delivery. This result was found to correspond with the other study conducted by Zhang et al.^31^. POSEIDON4 follow by POSEIDON2 showed the highest probability for PTD, PROM, and gestational diabetes as compared to other groups with statistical significance, and can be explained by the advanced maternal age^32^. However, when the finding was adjusted to BMI and age for the four groups, the differences did not reach statistical significance. Our results about early PTD and pre-eclampsia coincided with those of Sazonova et al.^28^, which increased in POSEIDON4. Placenta abruption was only noticed in POSEIDON2 and POSEIDON1, while POSEIDON3 reported a high rate for placenta previa. Finding some differences among previous studies can be partly explained by the methodology of measuring and comparing perinatal outcomes^33^.

Regarding optimistic and conservative CLBR, POSEIDON1 showed the highest in both in all three cycles followed by POSEIDON3, POSEIDON2, then POSEIDON4, respectively. This means that younger women supposedly with better ovarian reserve have a higher probability for live births as compared to older women supposedly with poor ovarian reserve. But Yuan Li et al*.* noted that the optimistic and conservative CLBR after three IVF/ICSI cycles were better in POSEIDON1, 2, 3 then 4, respectively^23^. We noticed that all optimistic CLBR values were higher than their corresponding conservative CLBR, this can explain by the decreasing of denominator when considering optimistic CLBR depending on the formula. Since from formula assumptions, optimistic over estimates while conservative underestimates CLBR, the true CLBR in each cycle is postulated to be lying along the recorded optimistic and conservative CLBR^34^. Further observation revealed little difference after three cycles between conservative and optimistic CLBR. The report in the literature on CLBR was significantly different based on BMI and age; low CLBR was found with the age of ≥ 42 years and obesity^35^. In all the four groups, CLBR increased from cycle one subsequently to cycle three, meaning despite their infertility status, low prognosis women can increase their probability of conception by undergoing multiple cycles of IVF/ICSI. These findings correspond with the literature, where studies founded that CLBR after one cycle was reported to be low in women with low prognosis depending on the Bologna criteria or POSEIDON criteria^23,36^, and a better CLBR after three cycles was indicated by Xu et al.^37^. Despite increasing CLBR from cycle one to cycle three, Yuan Li reported that the magnitude of increase decreased and chart plateaus as multiple subsequent ovarian stimulations exhausted antral follicles^23^.

The current study didn’t focus on the type of ovarian stimulation protocols, because the suitable stimulation protocols for patients with low prognosis were used depending on physician’s experiences and previous studies^38,39^. In addition, because another study didn’t notice that the changing protocol after the first IVF/ICSI cycle can improve the results^23^, but this point needs further research.

Despite our study’s intriguing results on the evidence of perinatal outcomes in women with low-prognosis based on POSEIDON classification, interpretations should be cautiously due to a number of possible biases encountered. Our study was a retrospective cohort and was associated with information, selection, and attrition biases. Others were unmatching of participants by ovarian stimulation protocol, the small sample size in some comparison groups, and combining fresh and frozen embryo participants despite the two having different perinatal outcomes^29^. Authors, utilized STROBE tool to mitigate reporting and publication biases in this study’s write-up. We call upon robust prospective studies, mitigating the aforementioned biases. We also suggest amendments to the current POSEIDON criteria, including the aforementioned confounding factors.

## Conclusion

Younger women with low prognosis with normal or diminished ovarian reserve (POSEIDON1 and 3) had an acceptable probability for live births and better perinatal outcomes compared to older women with normal ovarian reserve (POSEIDON2). Advanced age women with diminished ovarian reserve (POSEIDON4) had the lowest percentage for healthy live birth. Age of infertile women with low prognosis is therefore considered as a critical parameter in predicting the perinatal outcome and CLBR. Despite ovarian reserve status, young women with low prognosis can increase their probability of conception and get relatively higher CLBR by undergoing multiple cycles of IVF/ICSI.

## Methods

This was a retrospective comparative observational study, conducted at Xiangya hospital, China. We reviewed 825 IVF/ICSI cycle records of low prognostic infertile women attending our reproductive center between 1st of January 2011 and the 31st of December 2015.

### Participants

To arrive maximal effectiveness, we reviewed the files of infertile couples who underwent complete conventional ovarian stimulation cycles in IVF/ICSI with oocyte retrieved with the first cycle between 2011 and 2015. All the patients were followed up for the live birth outcome until December 2018.

### Inclusion criteria

This study considered eligible participants with (1) low prognosis patient (4–9 oocytes retrieved after a conventional ovarian stimulation protocol), (Conventional ovarian stimulation protocol was defined as use at least a dose of FSH 150 IU per day); (2) aged between 25–40 years; (3) having been diagnosed with primary or secondary infertility and have never been donated an ovum; (4) having recorded for AFC and AMH in the 3 months prior to ovarian stimulation; (5) have undergone control ovarian stimulation (COS) by any suitable protocols of IVF/ICSI used in our reproductive center, with corpus luteum support, fresh or frozen embryo transfer; (6) live birth (singleton, twin, or other multiples) pregnancies.

### Exclusion criteria

(1) Patient with a normal response for ovarian stimulation; (2) received ovum donor for IVF ; (3) not having received a trigger for final oocyte maturation; or embryo transfer ; (4) patients with oocyte freezing cycles; (5) women in any group who aged to become eligible for another group, during the follow-up; (6) Cases with severe male factors.

Accepted patients in the study were divided based on POSEIDON criteria to:

1. POSEIDON 1: < 35 years + normal ovarian reserve AFC ≥ 5 and AMH ≥ 1.2 ng/ml).
2. POSEIDON 2: ≥ 35 years + normal ovarian reserve (define as mentioned above).
3. POSEIDON 3: < 35 years + diminished ovarian reserve (AFC < 5 and AMH < 1.2 ng/ml).
4. POSEIDON4: ≥ 35 years + diminished ovarian reserve (define as mentioned above).

Thus eligible participants were followed up until 31st of December 2015 for any of the following to happen during censoring of a participant: (1) Completing the third cycle of IVF/ICS which also marked the end of our study follow up; (2) achieving a biochemical pregnancy or clinical singleton or twins pregnancy (defined by any registered for a single or more embryonic heartbeat at ultrasonography); (3) having a live birth (defined by delivery of a baby ≥ 28 weeks gestational age) in any of the cycles, also this marked the end of our study follow up; (4) abstaining from scheduled follow-up visits at our center during the period of our study.

All POSEIDON groups underwent at least one cycle of egg retrieval, the cycle marked by transfers of all fresh and/or frozen embryos resulting from one episode of ovarian stimulation.

The main outcomes were CLBR with perinatal and obstetric outcomes (Birthweight, gestational age, ectopic pregnancies, small for gestational age, low birth weight, large for gestational age, abortion, preterm delivery, Early PTD, malformations, premature rupture of membrane, pre-eclampsia, placental abruption, placenta previa, gestational diabetes, mode of delivery, malpresentation, and stillbirth) with CLBR per cycle after IVF/ICSI. And IVF/ICSI-technique outcomes: The number of (follicle ≥ 14 ml, oocytes retrieved, metaphase II Oocytes, embryos Obtained, top quality Embryos Obtained, and embryo transfer (Fresh and frozen)) as secondary outcomes.

### Variables

So our study had perinatal and obstetric outcomes after achieving a live birth including singleton pregnancy as well as CLBR per cycle including all embryo transfers (fresh and frozen-thawed) as our main outcome. And clinical pregnancy with biochemical pregnancy (defined by βHCG > 25mIU/mL) as additional outcomes.

In general, a good perinatal outcome considers as the delivery of live birth, normal-weight (≥ 2,500 g), term infant^40^, and we studied in this research the pregnancy outcomes of only a singleton pregnancy. The perinatal and obstetric outcomes in our study were: Birthweight, gestational age, ectopic pregnancies, small for gestational age (< 10%) (SGA), low birth weight (< 2,500 g) (LBW), large for gestational age (LGA) (> 90%) (≥ 4,500 g), abortion, preterm delivery (PTD) (< 37 weeks), Early PTD (< 32 weeks), malformations, premature rupture of membrane (PROM), pre-eclampsia, placental abruption, placenta previa, gestational diabetes, mode of delivery, malpresentation, and stillbirth (intrauterine death ≥ 28 gestational weeks). We calculated also demographic characteristics, obstetrics, and gynecological history at the start of the first cycle (menstrual cycle history, previous pregnancy history, infertility (including type, duration, and causes as female or male), baseline hormonal levels (AMH, FSH, LH, E2, and Progesterone in day 2–4 of the menstrual cycle), and AFC.

Regarding IVF/ICSI parameters, other continuous variables were drugs administered; dosages and durations of FSH, HMG, GnRH agonist, GnRH antagonist, human growth hormone (GH), and clomiphene citrate, with the duration of stimulation too.

All aforementioned variables were retrieved from patients’ medical records and were compared between the four POSEIDON groups identified women.

Sample size calculation: Using the following formula^41^ n = ((z)^2^ p (1 − p))/d^2^ where, n = the estimated sample size; z = the standard normal deviation for 95% confidence level set as 1.96; *p*—the estimated low prognosis of problem 13.09%^42^; q = 1-p; d = the precision error set as 5%

Substituting the values into the formula:

$$ {\text{n}} = \left[ {1.96^{2} \times 0.1309\left( {1 - 0.1309} \right)} \right]/\left( {0.05} \right)^{2} = 174.81 $$

Therefore, a minimum of 175 participants would need to be included.

Ovarian stimulation and oocyte retrieval protocol: Ovarian stimulation (OS) was individualized based on participant’s age, BMI, basal hormones (FSH, LH, E2) and AFC. Details (if any) of previous ovarian response was also considered. This was considered to be useful in avoiding Ovarian Hyperstimulation Syndrome (OHSS). Participants in all IVF/ICSI cycles received recombinant Follicle-stimulating hormone (rFSH), with one or more of these drugs: human menopausal gonadotrophins (HMG)^16^, either GnRH agonist (short or long-acting) or GnRH antagonist, Human growth hormone (GH) and Clomiphene Citrate (CC). Protocols utilized in our study were: (1) Mild stimulation (minimally stimulation); (2) Double stimulation protocol; (3) PPOS protocol (Progestin-primed Ovarian Stimulation); (4) long or short-acting GnRH agonist protocol or GnRH antagonist protocol. Serial transvaginal ultrasound examination monitoring of follicle growth and evaluations of serum LH, E2, and progesterone were continually been carried out during the ovarian stimulation to adjusting the dosage of rFSH. Human chorionic gonadotrophin (hCG medicament) 4,000–10,000 IU was administered when 2–3 follicles reached the size of 20 mm or higher. Oocyte retrieval was then performed, within 36 h using transvaginal ultrasonography-guided aspiration, to avoid low fertilization rates of oocytes retrieved from less mature follicles^43^. The fresh embryo transfers generally took place three days after the oocyte retrieval. In our clinic, no more than 2 cleavage embryo was transferred under the guidance of ultrasonography, but in the following cases, only one cleavage embryo was transferred: scare uterine, less height than 150 cm, cervical incompetence, and cardiovascular disease. And the same embryo transfer policy for a frozen embryo. Preimplantation genetic diagnosis (PGD) for inherited genetic diseases, was used to reduce the probability of transfer embryos with genetically abnormal when it was necessary^44,45^.

### Statistical analysis

Analyses in this study were conducted depending on the outcome of interest and the type of variables (dependent or independent variables). Categorical variables were expressed as percentages and compared by Pearson’s Chi-square and median test. Obstetrics and perinatal outcome were compared by their probability of events utilizing the total number of only singleton live birth as the denominator. Continuous variables comparison between the four groups were done either using the Analysis of variance (ANOVA) or Kruskal–Wallis test or Median test depending on whether the variables displayed, normal or non-normal distributions after conducting normality test by Shapiro–Wilk test. The odds ratio of obstetric and perinatal parameters was calculated as crude and adjusted to age and BMI. All statistical analyses were done utilizing a computer software SPSS (Version22) at a 95% significance level.

### Cumulative live birth rate calculation

CLBR was calculated per cycle because low prognosis women often choose to undergo many potentially risky (ovarian stimulation and oocytes retrieving) procedures of IVF/ICSI in order to bank embryos before utilization, and assuming that censored participants with low prognosis had not had a significant probability of having a live birth. This made it vital to specify beforehand the optimal and conservative estimates across and after a three complete IVF/ICSI treatment cycle were calculated because optimistic methods express that patients who did not return for subsequent IVF/ICSI cycles would have the same opportunity of a pregnancy resulting in a live birth as those who needed and continued the treatment; conservative methods assumed no live births for patients who did not continue.

The CLBR within each cycle is based on the estimate of the live birth rate^45,46^. In calculating optimistic CLBR the fraction-function had a number of women with at least one live birth (i.e. first delivery) achieved in fresh or frozen cycles, as the numerator and the total women, who attempted ovarian stimulation as part of IVF/ICSI and underwent ovum collection, as the denominator, while we used the formula, to calculate conservative CLBR per cycle 1, 2 and 3^21,47^, as demonstrated in Table S2 (Supplementary file). CLBR was compared between these groups after adjusting BMI, AMH, AFC, and basic hormonal analyses (FSH, LH, E2, and Progesterone).

### Ethical consideration

This study was approved by the Human Research Ethics Committee of the health service and The Institutional Review Board (IRB) of Xiangya Hospital, Central South University, and given approval number: 2018121155 date: 13/12/2018.

### Informed consent

Because this study was a retrospective observational study, data were obtained by utilizing patient records review of infertile women with low prognosis. The statement on informed consent was obtained from participants through phone calls or WeChat. Participants were assured that the study involved basic information and clinical data, and the data were to be used only for academic research and not for any commercial purpose. All authors confirm that the methods used in this study were carried out in accordance with relevant guidelines and regulations of the Human Research Ethics Committee of the health service and The Institutional Review Board (IRB) of Xiangya Hospital.

## Supplementary information

Supplementary information.

## Abbreviations

POSEIDON: Patient Oriented Strategies Encompassing Individualized Oocyte Number
ART: Assisted reproductive technology
IVF/ICSI: In-vitro fertilization
POR: Poor ovarian responders
CLBR: Cumulative live birth rates
AFC: Antral follicle count
AMH: Anti-Mullerian hormone
SPSS: Statistical Package for the Social Sciences
FSH: Follicle-stimulating hormone
LH: Luteinizing hormone
HMG: Human menopausal gonadotrophins
GnRH a: Gonadotropin releasing hormone agonist
GnRH A: Gonadotropin releasing hormone antagonist
GH: Human growth hormone
CC: Clomiphene citrate
BMI: Basal metabolic index
COS: Control ovarian stimulation
PGD: Preimplantation genetic diagnosis
