# Supplementary material for: Cumulative live-birth, perinatal and obstetric outcomes for POSEIDON groups after IVF/ICSI cycles: a single-center retrospective study
Source: Sci Rep. 2020 Jul 16;10:11822. doi: 10.1038/s41598-020-68896-1 (PMC7366673; doi:10.1038/s41598-020-68896-1)
Supplement: Supplementary file 1 — Supplementary information. [file 41598_2020_68896_MOESM1_ESM.docx]

**Cumulative live-birth, perinatal and obstetric outcomes for POSEIDON groups after IVF/ICSI cycles: a single-center retrospective study**

**Authors:** Raed K. Abdullah^1^ MD; Nenghui Liu ^1^ Ph.D; Yuhao Zhao^1^ Dr.; Yang Shuang^1^ Dr.; Zhang Shen^1^ Dr.; Hong Zeng^1^ MD.; Jielei Wu^1^ Dr.

**Affiliations:** ^1^Reproductive Medical Center, Xiangya Hospital, Central South University, Changsha city, Hunan province – 410008, People’s Republic of China**.**

**Correspondence should be addressed to Nenghui Liu, Email:** dr_liunenghui@126.com

Reproductive Medical Center, Xiangya Hospital of Central South University, 87 Xiangya Road, Changsha city, Hunan province – 410008, People’s Republic of China.

Tel: +861329740221

**Emails of all authors:**

Raed K. Abdullah: raedabdullah77@csu.edu.cn

Nenghui Liu: [dr_liunenghui@126.com](mailto:dr_liunenghui@126.com)

Yuhao Zhao: [535586816@qq.com](mailto:535586816@qq.com)

Yang Shuang: [2204120324@csu.edu.cn](mailto:2204120324@csu.edu.cn)

Zhang Shen: [2201130508@csu.edu.cn](mailto:2201130508@csu.edu.cn)

Hong Zeng: [minizenghong@126.com](mailto:minizenghong@126.com)

Jielei Wu: [Wujielei0224@163.com](mailto:Wujielei0224@163.com)

**Table S1** Perinatal and obstetric outcomes’ crude and adjusted odds ratios by age and body mass index

| **Perinatal/Obstetric outcome** | **POSEIDON 1** | | | **POSEIDON 2** | | | **POSEIDON 3** | | | **POSEIDON 4** | | |
| --- | --- | --- | --- | --- | --- | --- | --- | --- | --- | --- | --- | --- |
|  | **COR (CI)** | **AOR (CI)** | **p-Value (COR; AOR)** | **COR (CI)** | **AOR (CI)** | **p-Value (COR; AOR)** | **COR (CI)** | **AOR (CI)** | **p-Value (COR; AOR)** | **COR (CI)** | **AOR (CI)** | **p-Value (COR; AOR)** |
| **LBW** | 1.898 (0.386-9.326) | 1.038 (0.080-13.488) | 0.430;  0.977 | 1.337 (0.218-8.181) | 1.494 (0.237-9.395) | 0.754; 0.669 | 1.111 (0.153-8.070) | 0.645 (0.044-9.482) | 0.917; 0.749 | 1 | 1 | 0.824; 0.889 |
| **Mode of delivery** | 8.252 (3.604-18.894) | 19.036 (6.054-59.860) | **0.000;**  **0.000** | 3.072 (1.240-7.612) | 3.002 (1.202-7.502) | **0.015; 0.019** | 3.128 (1.225-7.984) | 6.848 (2.069-22.663 | **0.017; 0.002** | 1 | 1 | **0.000; 0.000** |
| **PTD (<37weeks)** | 3.544 (1.190-10.548) | 4.157 (.892-19.374) | 0.088; 0.168 | 2.340 (.708-7.735) | 2.066 (.619-6.894) | 0.163; 0.238 | 1.714 (.466-6.300) | 1.973 (.373-10.439) | 0.417; 0.424 | 1 | 1 | **0.023**;  0.070 |
| **Placenta previa** | 1.606 (0.165-15.660) | 4.436 (0.175-112.232) | 0.684;  0.366 | 2.703 (0.276-26.448) | 2.915 (0.290-29.334 | 0.393; 0.364 | 3.412 (0.348-33.464) | 9.225 (0.357-238.628) | 0.292; 0.181 | 1 | 1 | 0.663; 0.530 |
| **Gestational diabetes** | 1.066 (0.260-4.364) | 1.628 (0.163-16.291) | 0.929;  0.678 | 1.187 (0.259-5.447) | 1.121 (0.240-5.243) | 0.826; 0.884 | 1.112 (0.218-5.670) | 1.664 (0.149-18.541) | 0.898; 0.679 | 1 | 1 | 0.997; 0.979 |
| **Malpresentation** | 6.567 (0.792-54.423) | 8.007 (0.946-67.761) | 0.081; 0.056 | 9.274 (1.210-71.088) | 12.427 (1.062-145.470) | **0.032;**  **0.045** | 1.110 (0.068-18.029) | 1.469 (0.068-31.857) | 0.942; 0.806 | 1 | 1 | **0.041; 0.041** |
| **Singleton live birth (≥28w)** | 2.305 (1.33-3.99) | 4.137 (1.67-10.274) | **0.003**;  **0.002** | 1.815 (0.99-3.32) | 1.794 (0.972-3.310) | 0.053; 0.061 | 0.967 (0.50-1.89) | 1.671 (0.644-4.339) | 0.921; 0.291 | 1 | 1 | **0.003; 0.001** |
| **Biochemical pregnancy** | 2.302 (1.368-3.872) | 3.540 (1.421-8.815) | **0.002;**  **0.007** | 1.542 (0.875-2.716) | 1.520 (0.855-2.700) | **0.134; 0.153** | 1.298 (0.716-2.355) | 1.938 (0.777-4.839) | **0.390; 0.156** | 1 | 1 | **0.013; 0.019** |
| **Clinical pregnancy** | 2.154 (1.274-3.641) | 4.049 (1.639-10.000) | **0.004;**  **0.002** | 1.588 (0.883-2.856) | 1.633 (0.900-0.963) | **0.122; 0.107** | 0.853 (0.458-1.590) | 1.538 (0.612-3.866) | **0.617; 0.360** | 1 | 1 | **0.002; 0.001** |
| **Abortion/all cycles** | 0.745 (0.274-2.027) | 0.958 (0.165 | 0.564;  0.962 | 0.743 (0.241-2.298) | 0.830 (0.263 | 0.607; 0.751 | 0.778 (0.237-2.554) | 0.996 (0.162- | 0.679; 0.996 | 1 | 1 | 0.940; 0.990 |

**Key:** **COR** – Crude odds ratio; **AOR** – Adjusted odds ratio to age and body mass index; **CI** – Confidence interval; **LBW** – Low birth weight; **PTD** – Pre-term delivery; **BMI** – Body mass index; Bolded are statistically significant p-Values

**Table S2:**  The formula of CLBR

| **IVF/ICSI** | **Number of participants** | **Number of live births** | **Number of dropouts** | **Conservative CLBR** | **Optimistic CLBR** |
| --- | --- | --- | --- | --- | --- |
| **Cycle 1** | **N_1_** | **n_1_** | **-** | **n_1_/N_1_** | **n_1_/N_1_** |
| **Cycle 2** | **N_2_** | **n_2_** | **D_1_** | **(n_1_+n_2_)/N_1_** | **(n1+n2+(D1*n2/N2))/N1** |
| **Cycle 3** | **N_3_** | **n_3_** | **D_2_** | **(n_1_+n_2_+n_3_)/N_1_** | **(n1+n2+n3+(D1+D2)*n3/N3)/N** |

**Key:**

**N_1_, N_2_, N_3_** - Number of women entering treatment at cycles 1, 2 and 3 respectively.

**n_1_, n_2_, n_3_** - Number of women with at least one live birth in cycles 1, 2 and 3 respectively.

**D_1_** – Number of women dropped out after cycle 1 without having a live birth

**D_2_** – Number of women dropped out after cycle 2 without having a live birth
